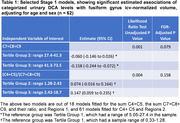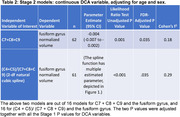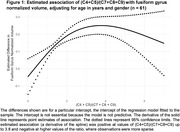# Urine dicarboxylic acid as a potential AD biomarker associated with fusiform gyrus volume in older cognitively healthy adults

**DOI:** 10.1002/alz.091892

**Published:** 2025-01-09

**Authors:** Alfred N Fonteh, Salvador Maldonado, Ryan Butler, Jimmy Kang, Thomas Macias, Anne Nolty, J Yu, Wendy J Mack, Helena C Chui, Yafa Minazad, Robert A Kloner, Xianghong Arakaki

**Affiliations:** ^1^ Huntington Medical Research Institutes, Pasadena, CA USA; ^2^ The Hill Medical Corporation, Pasadena, CA USA; ^3^ California Medical Business Services (CMBS), Pasadena, CA USA; ^4^ Fuller Theological Seminary, Pasadena, CA USA; ^5^ University of Southern California, Los Angeles, CA USA; ^6^ Department of Population and Public Health Sciences, Keck School of Medicine, University of Southern California, Los Angeles, CA USA; ^7^ Department of Neurology, Keck School of Medicine, University of Southern California, Los Angeles, CA USA; ^8^ Southern California Neurology Consultants, Pasadena, CA USA

## Abstract

**Background:**

Our study aims to determine if urine dicarboxylic acids (DCA) concentrations might be associated with specific brain region volumes in older cognitively healthy (CH) adults.

**Method:**

We recruited CH study participants (> 65 years) and acquired MRI data analyzed using NeuroQuant. Urine DCAs were quantified using GC‐MS. For n=62 participants, DCA variables of interest—C4+C5 and C7+C8+C9 (% of the sum of C4 to C10), and their ratio—were assessed for association with each of six intracranial volume (icv)‐normalized brain region volumes; C4+C5 with 61 additional regions. We modeled volume as a function of a discretized DCA variable (sample tertiles), age, and sex, for each pair. Pairs with a statistically significant estimated association were assessed by fitting natural cubic spline models with a range of spline degrees of freedom in a second stage of analysis. The Benjamini–Hochberg procedure was used to control the false discovery rate below q_1_ = 0.2 in the first stage and q_2_ = 0.05 in the second stage.

**Result:**

There was a significant estimated inverse association of categorical C7+C8+C9 with the fusiform gyrus (FG) volume (Table 1). Similarly, there was a significant estimated positive association between categorical (C4+C5)/(C7+C8+C9) and FG volume (Table 1). The FG was estimated to decrease by 0.004 for every one‐unit increase in C7+C8+C9, and this estimated association remained significant after adjusting P values for all tests (95% CI ‐0.007 to 0.002; adjusted P = .035, Table 2). The observed Cohen’s f2 for the DCA variable was 0.18. For the (C4+C5)/(C7+C8+C9)–FG couple, it was the 2‐df natural cubic spline model that had the lowest Bayesian information criterion values among the spline models considered, with a significant estimated association of the DCA variable with the FG (spline adjusted P = 0.035). Figure 1 depicts the estimated spline. The observed Cohen’s f2 for the spline was 0.29.

**Conclusion:**

Our analysis suggests that DCA metabolism may be associated with FG volume differences between individuals. Since the FG participates in visual processing, memory, and multisensory integration, urinary DCA levels may be biomarkers to monitor AD pathophysiology associated with differences in these functions.